# Pattern of Aeroallergen Sensitization and Quality of Life in Adult Thai Patients With Allergic Rhinitis

**DOI:** 10.3389/falgy.2021.695055

**Published:** 2021-11-15

**Authors:** Puspalal Katel, Bannapuch Pinkaew, Kanokporn Talek, Pongsakorn Tantilipikorn

**Affiliations:** Division of Rhinology and Allergy, Department of Otorhinolaryngology, Faculty of Medicine Siriraj Hospital, Mahidol University, Bangkok, Thailand

**Keywords:** allergic rhinitis, skin prick test, aeroallergen, quality of life, visual analog scale

## Abstract

The prevalence of allergic rhinitis (AR) is steadily rising in the Thai population, causing a major impact on the quality of life (QoL). Enhancing knowledge on common aeroallergens in the local setting helps in the appropriate prevention and management of AR. In this study, the demographic characteristics, clinical data, aeroallergen sensitization pattern, allergic symptoms, visual analog scale (VAS) score, and QoL are described. We evaluated the association between VAS, QoL, and severity of symptoms, except the aeroallergen sensitization pattern. We retrospectively reviewed the medical records of adult AR patients with a positive skin prick test (SPT) for at least one aeroallergen from January 2018 to May 2020. Standard descriptive and inferential statistics were used for analysis. A total of 366 patients were enrolled. Indoor aeroallergen sensitization and outdoor aeroallergen sensitization were observed in 32% and 7.9% of patients, respectively. Mono-sensitization was noted in 16.9% of patients, while poly-sensitization was noted in 83.1% of patients. Mites (65%) and sedge (39.3%) were the most common indoor and outdoor allergens. Nasal obstruction (74.6%), runny nose (63.7%), and nasal itchiness (61.5%) were the primary symptoms affecting the QoL. The association between VAS and symptom scores showed a trend of association with AR severity (Allergic Rhinitis and its Impact on Asthma [ARIA] classification) and VAS. AR has a significant effect on QoL in all domains of the validated generic (short-form-36, SF-36) and specific (rhino-conjunctivitis QoL questionnaire, Rcq-36) questionnaires. Mite and sedge remain the most common indoor and outdoor aeroallergens. The pattern of sensitization and number of aeroallergens were not associated with AR based on the ARIA guidelines. Meanwhile, symptoms of patients affected the QoL and VAS scores, which can be used as a quick and reliable tool for monitoring and stepping up or stepping down the treatment according to the next-generation guidelines. AR has a significant impact on the QoL of adult Thai patients.

## Introduction

Allergic rhinitis (AR) is an immunoglobulin (Ig) E-mediated inflammation of the nasal mucosa induced after allergen exposure and has three cardinal symptoms: sneezing, nasal obstruction, and rhinorrhoea (1). AR may also be frequently associated with asthma, as emphasized by the Allergic Rhinitis and its Impact on Asthma (ARIA) document (2). The skin prick test (SPT) is considered a standard diagnostic method because of its accuracy, reproducibility, and affordability; internationally, it remains the most acceptable and cost-effective means of diagnosing AR (3, 4). The effective management of AR requires a precise diagnosis, which includes the identification of IgE-mediated inflammation (4). The optimal management includes adequate control of symptoms through the provision of patient education, environmental control, and the use of pharmaceutical therapies and immunotherapy (5).

Allergic rhinitis represents a global health problem, affecting 10–20% of the population. The prevalence of AR is steadily rising in Thailand, with no signs of abating. According to a previous study, the prevalence of AR increased from 37.9 to 50.6% (6). AR has a major impact on the physical, mental, and social functioning of Thai patients. AR impairs work, sleep, and emotional health. With its increasing incidence and the inadequate control of symptoms, AR causes a socioeconomic burden (6). The pattern of aeroallergen sensitization varies according to the geographical region due to differences in climate, urbanization, and lifestyle. However, the sensitization pattern constantly changes with the changes in economic conditions, level of industrialization, and lifestyle, due to the alterations in the prevailing circulating aeroallergens. Knowledge of the up-to-date data regarding the offending aeroallergen in a local setting at a particular time is important for the effective management of AR (3, 7).

According to recently published 19-year (1998–2017) data from an ENT allergy clinic, Faculty of Medicine Siriraj Hospital, the mite *Dermatophagoides pteronyssinus* (*Dp*) remains the most common indoor aeroallergen, while sedge remains the most common outdoor aeroallergen among the Thai population (4). This study aimed to analyse the pattern of aeroallergen sensitization and describe the demographic and clinical data related to AR. It also aimed to describe the quality of life (QoL) and determine the association between QoL and the severity of AR symptoms. The short-form-36 (SF-36) and rhino-conjunctivitis QoL questionnaire (Rcq-36) questionnaires are generic and disease-specific QoL questionnaires, which have been translated and validated to be used in the Thai population (8, 9). Thus, the present study assessed the impact of AR in Thai patients using the SF-36 and Rcq-36 questionnaires.

## Materials and Methods

This study was a retrospective chart review of adult patients diagnosed with AR by a positive SPT for at least one aeroallergen, conducted at the Rhinology and Allergy Unit, Department of Otorhinolaryngology, Faculty of Medicine Siriraj Hospital, Mahidol University, between January 2018 and May 2020. Patients who were unable to completely fill out the case record forms or QoL questionnaires were excluded from our analysis. The following demographic and clinical data were obtained: age, gender, presenting symptoms, duration of symptoms, SPT results for various aeroallergens, the score of each domain in SF-36 (generic QoL questionnaire), the score of each domain in the Rcq-36 (specific questionnaire), and classification and severity of AR based on the ARIA guidelines. The patients were asked to discontinue taking oral antihistamines for 7 days prior to the test day. The visual analog scale (VAS) was used to determine the severity of AR symptoms (0–10 cm), and a diary card was provided to record the patient's symptoms for the last 7 days prior to the SPT.

The SPT was performed on the ventral aspect of the forearm by placing one drop of each allergenic extract 3-cm apart; then, the skin was pricked with a 26-gauge separate disposable needle in the middle of each allergen drop, using light pressure. The result was considered positive if the wheel diameter was larger than 3 mm and had an accompanying flare. Siriraj Allergen Vaccine (SAV), which contains standardized allergen extracts and has proven allergenic potency, was used (4, 10). Allergen extracts from mite *Dp*, American cockroach (*Periplaneta americana*), cats, dogs, molds (*Aspergillus* spp., *Penicillium* spp., and *Cladosporium* spp.), Bermuda grass (*Cynodon dactylon*), para grass (*Brachiaria mutica*), sedge (*Cyperaceae*), careless weed (*Amaranthus palmeri*), and Kapok (*Ceiba pentandra*) were used in our center. Histamine and normal saline were used as positive and negative controls, respectively (4). All SPTs were performed by qualified technicians.

Validated Thai versions of the SF-36 (8) and Rcq-36 questionnaires (9, 11) were used in this study. The AR patients in our center were instructed to draw a cross on the horizontal line of the VAS at the specific point that most accurately indicated their symptom severity (from 0 to 10 cm). The symptom diary card is a form that contains a list of AR-related symptoms. Patients were asked to recollect the severity of the given symptom and provide a score accordingly. The list of symptoms provided by the study patients included an itchy nose, sneezing, nasal obstruction, runny nose, post-nasal drip, loss of smell, itchy eyes, and others. Each symptom was scored from 0 to 3: 0 = none, 1 = mild, 2 = moderate, or 3 = severe. The total nasal symptom score (TNSS) is the sum of scores for nasal congestion, sneezing, nasal itching, and rhinorrhoea at each time point. TNSS was calculated by adding the score for each symptom, with a total score of 12 points (12).

Data processing and analysis were performed using PASW Statistics (SPSS Inc., Chicago, IL, USA). The data were expressed as frequency and percentage for categorical data or mean ± SD for continuous data. Normally distributed continuous data were evaluated using the one-sample Kolmogorov-Smirnov test. The data were classified according to the ARIA guidelines. The differences in the type of aeroallergen and type of sensitization among the AR severity groups (ARIA classification) were determined by chi-square tests. The association between TNSS, VAS, and QoL scores in the SF-36 and Rcq-36 questionnaires and type of aeroallergen, pattern of sensitization, or ARIA classification was analyzed using the Kruskal-Wallis test; meanwhile, the association of TNSS, VAS, and pattern of sensitization was determined using the Mann-Whitney *U*-test for non-normally distributed data and using unpaired *t*-tests and ANOVA for normally distributed data. Clinical symptoms and the pattern of sensitization were evaluated for association with severity and QoL. The factors associated with the pattern of sensitization and ARIA classification with a p-value <0.05 using univariate analysis by stepwise method and multivariate analysis are shown as *p*-value and odds ratio (OR) with 95% CI. A *p* value < 0.05 was considered significant.

This study was approved by the Siriraj Hospital Institutional Review Board (approval number: **Si 296/2019**).

## Results

The patient demographic and clinical characteristics are shown in Table 1. A total of 366 patients who fulfilled the inclusion criteria were included in our study. Among them, 236 (64.5%) were women and 130 (34.5%) were men. The mean age of the female participants was 35.7 ± 12.4 years, while that of the male participants was 33.2 ± 12.6 years. The mean duration of symptoms was 9.7 ± 10.1 years in women and 9.0 ± 7.8 years in men. The mean ages of onset of AR were 25.5 ± 13.6 years in women and 23.3 ± 13.4 years in men.

**Table 1:** Demographic and clinical characteristics of patients (*n* = 366).

**Characteristic**	**Value**
**Sex [*****n*** **(%)]**	
Female	236 (64.5%)
Male	130 (35.5%)
**Age (mean ± SD)**	
Female	35.7 ± 12.4
Male	33.2 ± 12.6
**Duration of symptom(year) (mean + SD)**	
Female	9.7 ± 10.1
Male	9.0 ± 7.8
**Age of onset (year) (mean ± SD)**	
Female	25.5 ± 13.6
Male	23.3 ± 13.4
**Type of aeroallergen [*****n*** **(%)]**	
Indoor	117 (32%)
Outdoor	29 (7.9%)
Both	220 (60.1%)
**Type of sensitization [*****n*** **(%)]**	
Mono-sensitization (one allergen)	62 (16.9%)
Poly-sensitization (more than one allergen)	304 (83.1%)
**Symptoms [*****n*** **(%)]**	
Nasal obstruction	273 (74.6%)
Runny nose	233 (63.7%)
Nasal itchiness	225 (61.5%)
Sneezing	185 (50.5%)
Itchy eyes	183 (50%)
Cough	95 (26%)
Excessive tearing	78 (21.3%)

Poly-sensitization was observed in 304 (83.1%) patients, while mono-sensitization was observed in 62 (16.9%) patients. Sensitization to indoor aeroallergens was noted in 117 (32%) patients, while sensitization to outdoor allergens was noted in 29 (7.9%) patients. Sensitization to both the aeroallergens was observed in 220 (60.1%) patients. Nasal obstruction (74.6%), runny nose (63.7%), and nasal itchiness (61.5%) were the most common symptoms observed in AR patients in this study. These symptoms were frequently observed in patients with moderate-to-severe intermittent and persistent AR. Based on the ARIA classification, a majority of the patients had moderate-to-severe persistent AR (54%), followed by moderate-to-severe intermittent AR (22%), mild intermittent AR (14%), and mild persistent AR (10%) (Table 2).

**Table 2:** Type of aeroallergen, type of sensitization, symptom score, and VAS score classified based on the ARIA guideline (*n* = 366).

	**Mild** **intermittent**	**Moderate to** **severe intermittent**	**Mild** **persistent**	**Moderate to** **severe persistent**	***p*-value**
	**(*n =* 51)**	**(*n =* 82)**	**(*n =* 35)**	**(*n =* 198)**	
**Type of aeroallergen [*****n*** **(%)]**					
Indoor	19 (16.2)	20 (17.1)	10 (8.5)	68 (58.1)	*0.54*
Outdoor	5 (17.2)	5 (17.2)	2 (6.9)	17 (58.6)	
Both	27 (12.3)	57 (25.9)	23 (10.5)	113 (51.4)	
**Type of sensitization [*****n*** **(%)]**					
Mono-sensitization	10 (16.1)	11 (17.7)	4 (6.5)	37 (59.7)	*0.49*
Poly-sensitization	41 (13.5)	71 (23.4)	31 (10.2)	161 (53.0)	
**Symptom score (0–21) (mean ± SD)**					
Nasal itchiness	2.2 ± 3.0	3.0 ±4.0	3.9 ± 5.3	6.0 ± 5.8	*<0.001*
Sneezing	2.7 ± 3.0	3.7 ±3.5	5.0 ± 5.4	7.0 ± 5.7	*<0.001*
Nasal obstruction	2.9 ± 3.5	4.9 ±5.1	7.4 ± 6.2	10.0 ± 6.3	*<0.001*
Runny nose	2.0 ±2.7	3.8 ± 4.0	4.2 ± 4.5	6.6 ± 5.9	*<0.001*
Post-nasal drip	1.8 ± 3.5	3.1 ± 4.6	5.2 ± 6.4	5.5 ± 5.9	*<0.001*
Smell loss	2.0 ±4.4	2.1 ± 4.4	2.7 ± 5.0	3.0 ± 5.5	*0.003*
Itchy eyes	1.3 ±2.6	3.4 ± 4.9	3.9 ± 5.4	5.3 ± 6.1	*0.002*
**VAS (0–10) (mean ± SD)**	2.3 ± 1.4	4.8 ± 2.3	3.4 ± 2.3	6.1 ± 2.0	*<0.001*

*p < 0.05: Significant difference between the type of aeroallergen and type of sensitization with ARIA classification using the chi-square test*.

*p < 0.05: Significant difference between symptom scores and VAS with ARIA classification using the independent-samples Kruskal-Wallis Test*.

*VAS, visual analog scale; ARAI, Allergic Rhinitis and its Impact on Asthma*.

**Figure 1:**
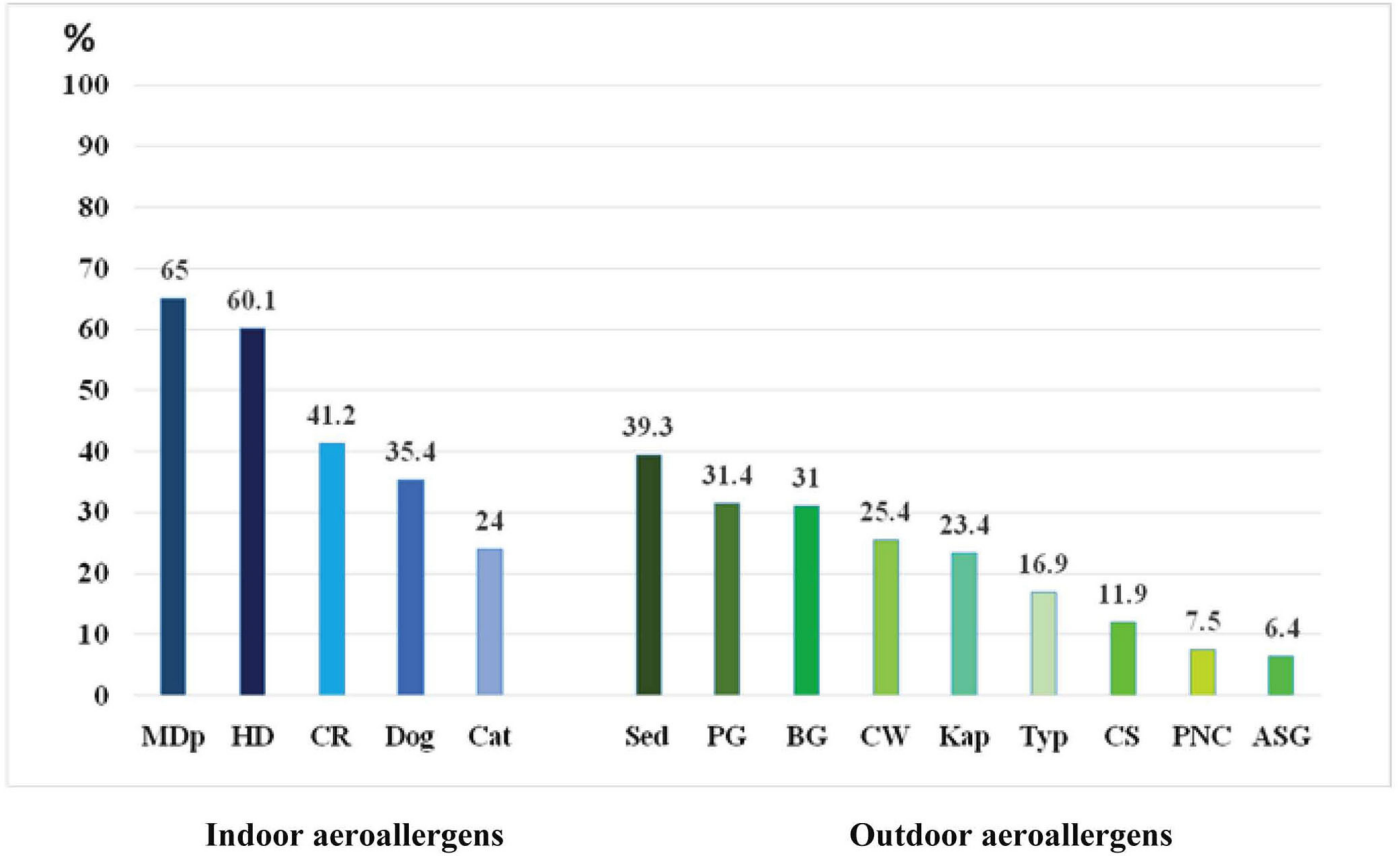
Pattern of aeroallergen sensitization (*n* = 366). Abbreviations: MDp, mite/*Dermatophagoides pteronyssinus*; HD, house dust; CR, cockroach; Sed, sedge; PG, para grass; BG, bermuda grass; CW, careless weed; Kap, Kapok; Typ, *Typha*; CS, *Cladosporium*; PNC, *Penicillium*; ASG, *Aspergillum*.

The frequency of sensitization to various aeroallergens, from January 2018 to May 2020, is shown in Figure 1. The indoor aeroallergen was most frequently sensitized against the mite *Dp* (65%) followed by house dust (60.1%), cockroach (41.2%), dog (35.4%), and cat (24%). Meanwhile, the outdoor aeroallergen was most frequently sensitized against sedge (39.3%) followed by para grass (31.4%), Bermuda grass (31%), careless weed (25.4%), Kapok (23.4%), *Typha* (16.9%), *Cladosporium* (11.1%), *Penicillium* (7.5%), and *Aspergillus* (6.4%) (Table 2).

**Figure 2:**
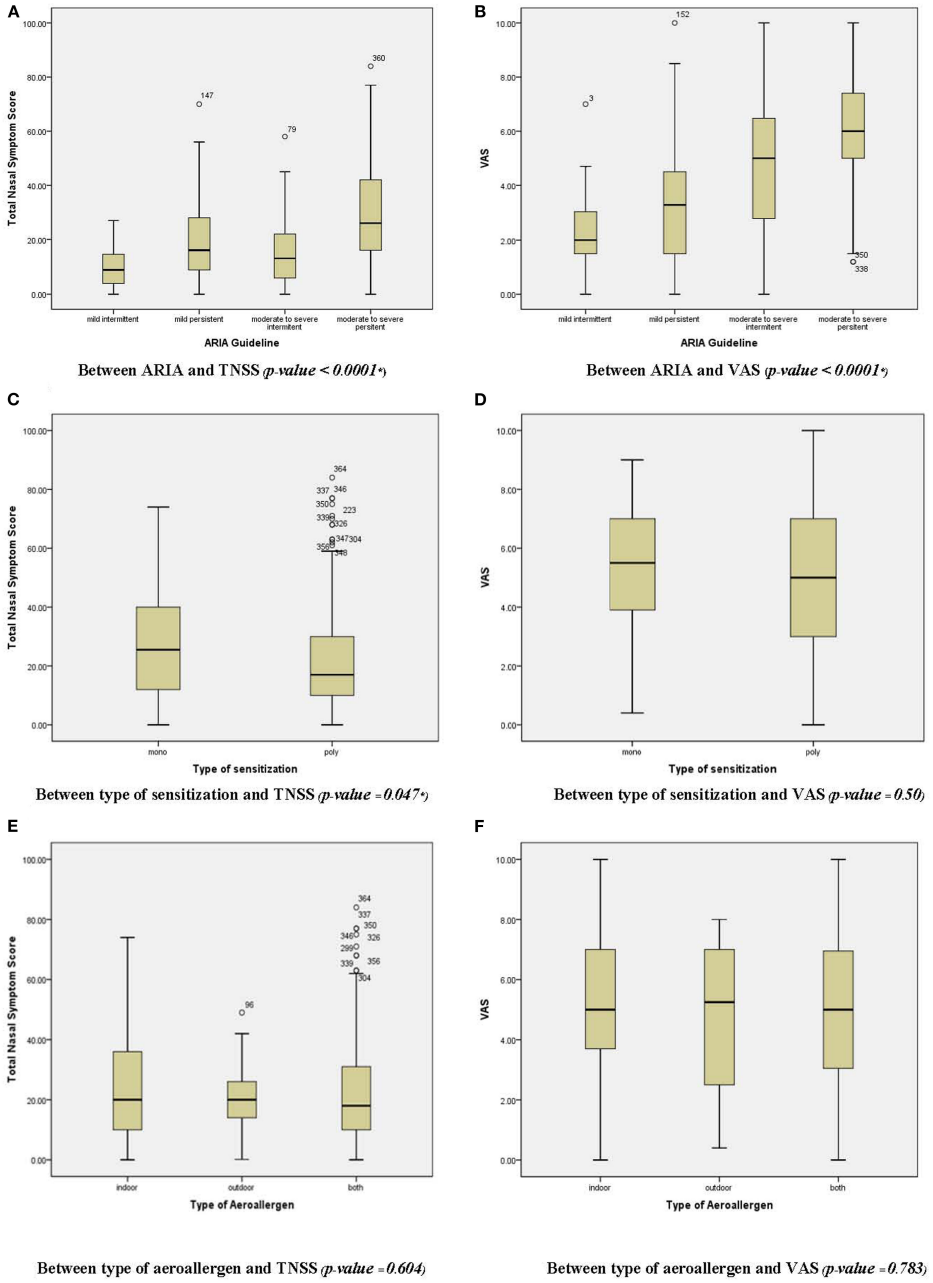
Association among TNSS, VAS score, ARIA classification, and type of sensitization and aeroallergen. TNSS, the total nasal symptom score; VAS, visual analog scale; ARAI, Allergic Rhinitis and its Impact on Asthma. *p < 0.05.

**Figure 3:**
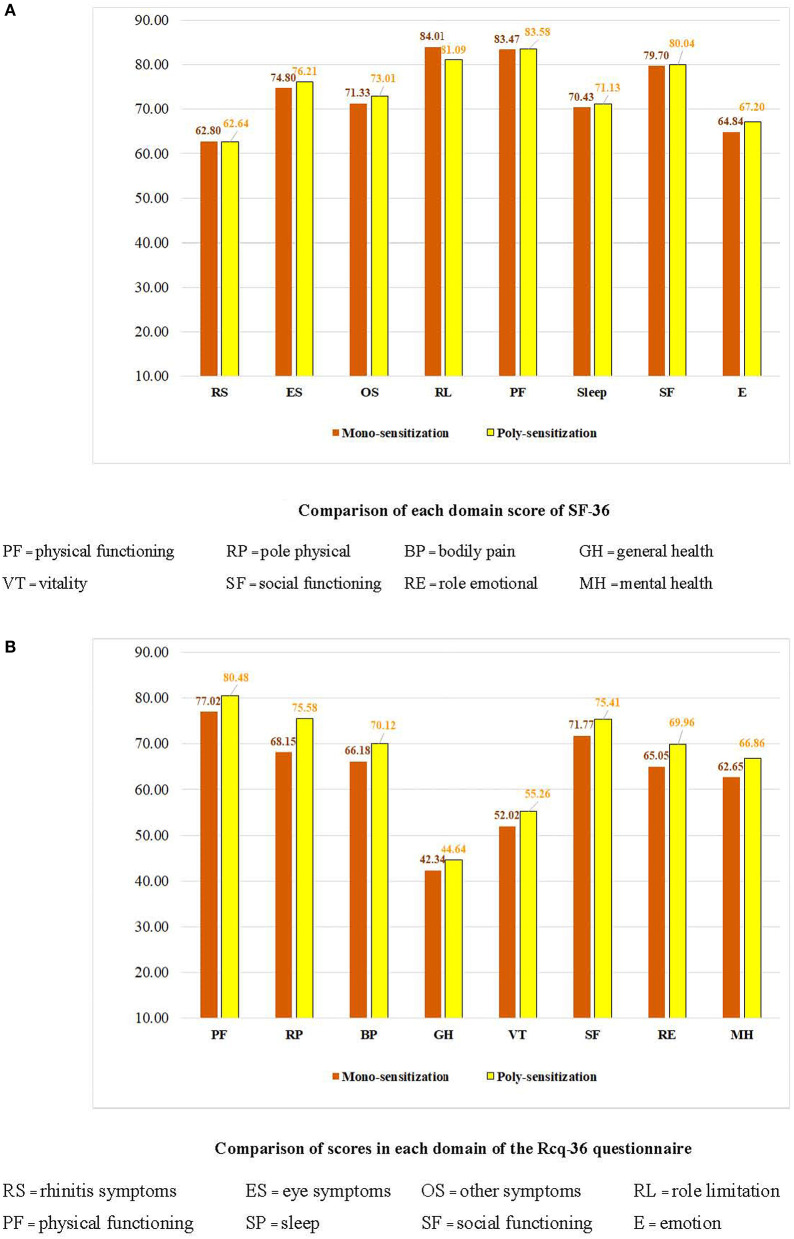
Comparison of QoL, SF-36, and Rcq-36 scores among types of aeroallergens. QoL, quality of life; SF, social functioning; Rcq, rhino-conjunctivitis QoL questionnaire; RS, rhinitis symptoms; ES, eye symptoms; OS, other symptoms; PF, physical functioning; SP, sleep; E, emotion. (*p* < 0.05: Significant difference between the type of aeroallergen and each domain of the SF-36 and Rcq-36 questionnaires using unpaired *t*-test).

**Figure 4:**
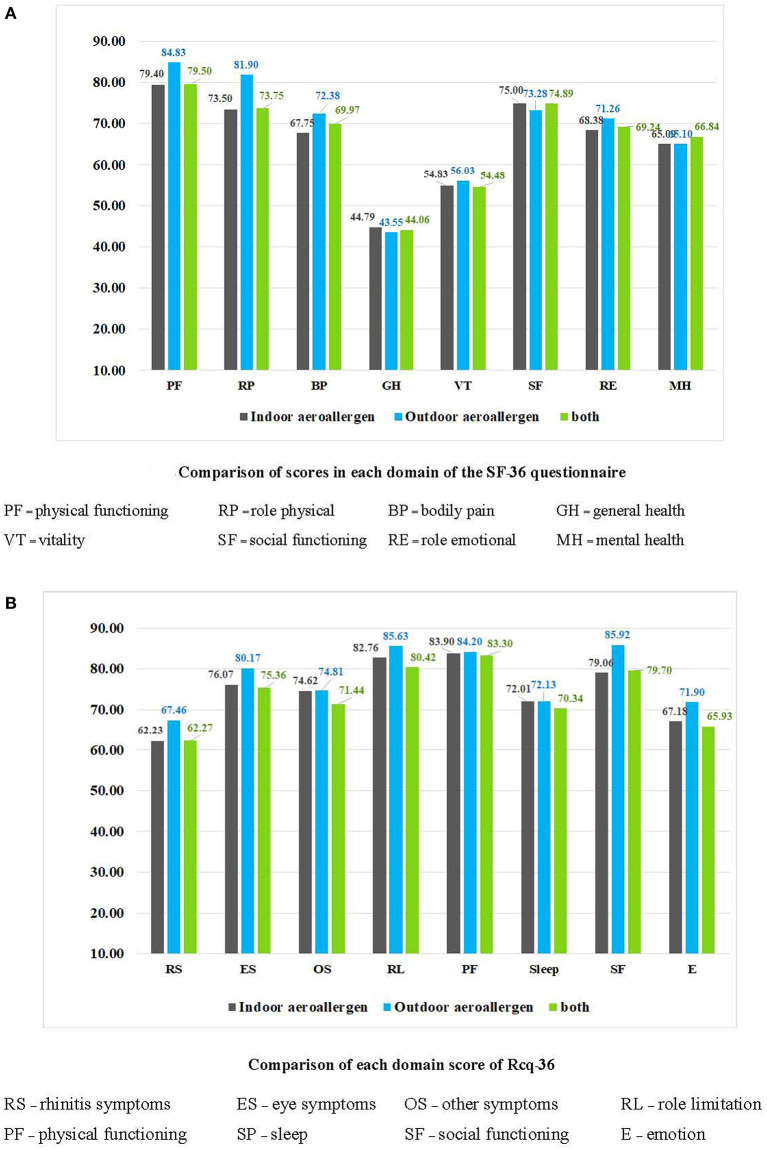
Comparison of QoL, SF-36, and Rcq-36 scores among the different types of sensitizations. QoL, quality of life; SF, social functioning; Rcq, rhino-conjunctivitis. (*p* < 0.05: Significant difference between the type of sensitization and each domain of the SF-36 and Rcq-36 questionnaires using analysis of variance).

**Figure 5:**
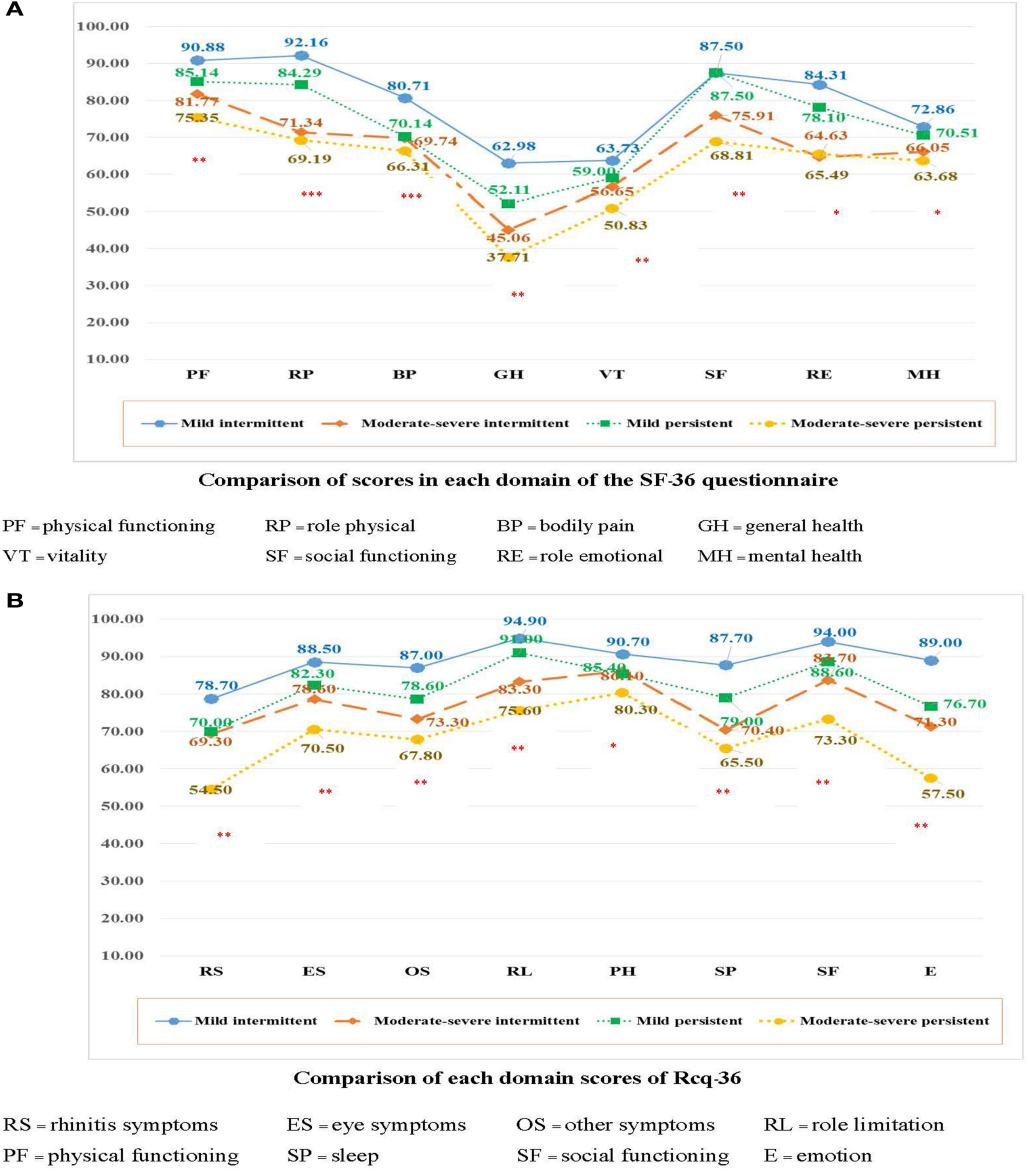
Comparison of QoL between the ARIA classifications (36). QoL, quality of life; ARIA, Allergic Rhinitis and its Impact on Asthma. (*p < 0.05, **p < 0.001: Significant difference among the ARIA classifications using analysis of variance).

Regarding the determinants of AR severity, a chi-square test revealed no significant relationship between the type of aeroallergen (*p* = 0.54) and the type of sensitization (*p* = 0.49) (Table 2). However, a significant difference was observed between the symptom score, VAS score, and ARIA classification using the independent-sample Kruskal-Wallis test (*p*< *0.05*). TNSS is significantly associated with the type of sensitization (Figure 2). An increased VAS score was associated with symptom severity and ARIA classification. In contrast, no significant association was found between the type of aeroallergen, sensitization, and the SF-36 and Rcq-36 domains (Tables 3, 4). Factors associated with the pattern of sensitization and ARIA classification are shown in Table 5. There was no association between the pattern of sensitization and patient's characteristics and symptoms. However, there was association between ARIA classification, symptom severity (TNSS and VAS), and QoL (Table 6). Figures 3, 4 show a comparison of the types of aeroallergens and sensitization among the SF-36 and Rcq-36 domains. Furthermore, when each domain of the generic SF-36 questionnaire was compared with the ARIA classification using ANOVA, a significant difference was observed (*p* < 0.05). The QoL in all domains was poorer as the severity of AR increases. Bodily pain, general health, and vitality were the most severely affected domains. The disease-specific QoL questionnaire (Rcq-36) also yielded poorer QoL in all domains, especially for rhinitis symptoms (54.5%), sleep (65.5%), and emotions (57.5%), with ANOVA yielding significant results (*p* < 0.05; Figure 5).

**Table 3:** Type of aeroallergen and each domain of the SF-36 and Rcq-36 questionnaires (*n* = 366).

**Domains**	**Type of aeroallergen**		***p*-value**
	**Mono-sensitization**	**Poly-sensitization**	
	**(*n =* 62)**	**(*n =* 304)**	
**SF-36**			
Physical functioning (PF)	77.02 ± 20.37	80.48 ± 20.31	*0.23*
Role physical (RP)	68.15 ± 41.03	75.58 ± 35.32	*0.14*
Bodily pain (BP)	66.18 ± 21.33	70.12 ± 19.42	*0.18*
General health (GH)	42.34 ± 19.98	44.64 ± 20.07	*0.41*
Vitality (VT)	52.02 ± 15.46	55.26 ± 17.96	*0.15*
Social functioning (SF)	71.77 ± 23.51	75.41 ± 21.99	*0.27*
Role emotional (RE)	65.05 ± 43.70	69.96 ± 38.82	*0.38*
Mental health (MH)	62.65 ± 16.58	66.86 ± 16.81	*0.72*
**Rcq-36**			
Rhinitis symptoms (RS)	62.80 ± 22.25	62.64 ± 22.85	*0.96*
Eye symptoms (ES)	74.80 ± 19.86	76.21 ± 21.22	*0.63*
Other symptoms (OS)	71.33 ± 21.13	73.01 ± 19.90	*0.55*
Role limitation (RL)	84.01 ± 16.82	81.09 ± 22.17	*0.24*
Physical functioning (PF)	83.47 ± 19.29	83.58 ± 20.66	*0.97*
Sleep (sleep)	70.43 ± 31.02	71.13 ± 29.04	*0.86*
Social functioning (SF)	79.70 ± 24.87	80.04 ± 25.74	*0.92*
Emotions (E)	64.84 ± 24.58	67.20 ± 26.90	*0.52*

*p < 0.05: Significant difference between the type of aeroallergen and each domain of the SF-36 and Rcq-36 questionnaires using unpaired t-test. Req, rhino-conjunctivitis QoL, questionnaire*.

**Table 4:** Type of sensitization and each domain of the SF-36 and Rcq-36 questionnaires (*n* = 366).

**Domains**	**Type of sensitization**			***p*-value**
	**Indoor** **aeroallergen** **(*n =* 117)**	**Outdoor** **aeroallergen** **(*n =* 29)**	**Both groups** **(*n =* 220)**	
**SF-36**				
Physical functioning (PF)	79.40 ± 20.81	84.83 ± 12.78	79.50 ± 20.86	*0.22*
Role physical (RP)	73.50 ± 36.74	81.90 ± 33.34	73.75 ± 36.64	*0.14*
Bodily pain (BP)	67.75 ± 21.44	72.38 ± 18.33	69.97 ± 19.04	*0.15*
General health (GH)	44.79 ± 19.74	43.55 ± 19.15	44.06 ± 20.40	*0.41*
Vitality (VT)	54.83 ± 17.47	56.03 ± 15.38	54.48 ± 17.98	*0.19*
Social functioning (SF)	75.00 ± 23.56	73.28 ± 20.52	74.89 ± 21.86	*0.24*
Role emotional (RE)	68.38 ± 39.60	71.26 ± 41.52	69.24 ± 39.64	*0.38*
Mental health (MH)	65.09 ± 18.39	65.10 ± 16.17	66.84 ± 16.06	*0.07*
**Rcq-36**				
Rhinitis symptoms (RS)	62.23 ± 21.98	67.46 ± 18.70	62.27 ± 23.59	*0.96*
Eye symptoms (ES)	76.07 ± 19.92	80.17 ± 17.36	75.36 ± 21.95	*0.63*
Other symptoms (OS)	74.62 ± 19.86	74.81 ± 18.57	71.44 ± 20.39	*0.55*
Role limitation (RL)	82.76 ± 18.56	85.63 ± 15.41	80.42 ± 23.31	*0.33*
Physical functioning (PF)	83.90 ± 19.78	84.20 ± 20.45	83.30 ± 20.82	*0.97*
Sleep (sleep)	72.01 ± 29.57	72.13 ± 29.65	70.34 ± 29.29	*0.86*
Social functioning (SF)	79.06 ± 26.34	85.92 ± 18.24	79.70 ± 25.95	*0.92*
Emotions (E)	67.18 ± 26.05	71.90 ±22.69	65.93 ± 27.22	*0.52*

*p < 0.05: Significant difference between the type of sensitization and each domain of the SF-36 and Rcq-36 questionnaires using analysis of variance. Req, rhino-conjunctivitis; QoL, questionnaire*.

**Table 5:** Factors associated with the pattern of sensitization and ARIA classification.

**Factor**	**Univariate analysis**		
	**Type of sensitization**	**Type of aeroallergen**	**ARIA classification**
	***p-value***	***p-value***	***p-value***
Age	*0.77*	*0.66*	*0.88*
Duration of symptom	*0.94*	0.04^*^	*0.88*
Age of onset	*0.76*	0.04^*^	*0.66*
Nasal itchyness	0.05^*^	*0.34*	<0.001^**^
Sneezing	*0.69*	*0.24*	<0.001^**^
Obstruction	*0.1*	*0.88*	<0.001^**^
Runny nose	*0.83*	*0.07*	<0.001^**^
Itchy eyes	*0.11*	*0.41*	<0.001^**^
**TNSS**	*0.15*	*0.18*	<0.001^**^
**VAS**	*0.27*	*0.47*	<0.001^**^
**SF-36**			
Physical functioning (PF)	*0.13*	*0.18*	<0.001^**^
Role physical (RP)	*0.29*	*0.4*	<0.001^**^
Bodily pain (BP)	*0.06*	*0.15*	<0.001^**^
General Health (GH)	*0.73*	*0.96*	<0.001^**^
Vitality (VT)	*0.18*	*0.7*	<0.001^**^
Social functioning (SF)	*0.19*	*0.98*	<0.001^**^
Role emotional (RE)	*0.2*	*0.66*	0.01^*^
Mental health (MH)	0.03^*^	*0.48*	<0.001^**^
**Rcq-36**			
Rhinitis ymptoms (RS)	*0.63*	*0.72*	<0.001^**^
Eye symptoms (ES)	*0.22*	*0.23*	<0.001^**^
Other symptoms (OS)	*0.21*	*0.27*	<0.001^**^
Role limitation (RL)	*0.81*	*0.24*	<0.001^**^
Physical functioning (PF)	*0.68*	*0.85*	<0.001^**^
Sleep (sleep)	*0.87*	*0.87*	<0.001^**^
Social functioning (SF)	*0.85*	*0.43*	<0.001^**^
Emotions (E)	*0.19*	*0.22*	<0.001^**^

* and ** indicates significant differences between the pattern of sensitization including type of sensitization, type of aeroallergen and ARIA classification and factors.

* *indicates p < 0.05 and*

** * indicates p < 0.001. VAS, visual analog scale; ARAI, Allergic Rhinitis and its Impact on Asthma; TNSS, the total nasal symptom score*.

**Table 6:** Independent risk ration of univariable and multivariable analysis by multinomial logistic regression.

	**ARIA Classification**					
	**Moderate to severe intermittent**		**Mild persistent**		**Moderate to severe persistent**	
**Univariable analysis**	**Crude Odds ratio (95% CI)**	***p-value***	**Crude Odds ratio (95% CI)**	***p-value***	**Crude Odds ratio (95% CI)**	***p-value***
Itchy eye	1.16 (1.04, 1.29)	0.01^*^	1.18 (1.045, 1.33)	0.007^*^	1.23 (1.11, 1.37)	<0.0001^**^
TNSS	1.06 (1.02, 1.11)	0.002^*^	1.10 (1.05, 1.15)	<0.0001^**^	1.14 (1.09, 1.18)	<0.0001^**^
VAS	2.02 (1.59, 2.58)	<0.0001^**^	1.45 (1.11, 1.90)	<0.0001^**^	2.74 (2.14, 3.50)	<0.0001^**^
**Multivariable analysis** **(Enter method)**	**Adjusted Odds ratio (95% CI)**	***p-value***	**Adjusted Odds ratio (95% CI)**	***p-value***	**Adjusted Odds ratio (95% CI)**	***p-value***
Itchy eye	1.02 (0.90, 1.16)	<0.0001^**^	1.06 (0.93, 1.21)	*0.401*	1.00 (0.89, 1.13)	*0.977*
TNSS	1.01 (0.96, 1.06)	*0.8849*	1.06 (1.00, 1.11)	0.045^*^	1.08 (1.03, 1.13)	0.002^*^
VAS	1.20 (1.54, 2.59)	<0.0001^**^	1.24 (0.93, 1.67)	*0.159*	2.35 (1.81, 3.05)	<0.0001^**^

* and ** indicates significant differences ARIA classification and itchy eye, TNSS and VAS.

* *indicates p-value was < 0.05 and*

** *indicates p-value was < 0.001*.

## Discussion

The Mite was the most common sensitizing indoor aeroallergen (65%), while sedge remained the most common sensitizing outdoor aeroallergen (39.3%) reported in our study, which was conducted from January 2018 to May 2020. When compared to the 19-year (1998–2017) data from the same center (4), no significant changes in the distribution of sensitization patterns were observed. However, the percentage of sensitization against mites increased from 54.8 to 65%, while that of sensitization against cockroaches increased from 36 to 41.2%. There was no significant change in the percentage sensitization pattern in the outdoor aeroallergen group. Sedge, para grass, and Bermuda grass were the top three outdoor aeroallergens that caused sensitization in our study; this finding is similar to those of a previous study conducted in the same center. This result indicates that both indoor and outdoor allergens remain the cause of AR among Thai patients. A study from another tertiary care hospital in Bangkok reported a mite *Dp* sensitization rate of 50.1% within a 12-year period (from January 2004 to December 2015) (13), which establishes the fact that there is an upwards trend in mite sensitization in adult Thai AR patients.

House dust mite is the most common sensitizing aeroallergen worldwide and causes sensitization in up to 90% of Asian atopic patients (7, 14). The mite sensitization rates were 57.5% in South coast China (15), 97.4% in the Philippines (7), 68.5% in Singapore (16), 21.4% in South Korea (17), and 63% in Hong Kong (3). The aeroallergen sensitization pattern in Thai adults is consistent with the findings from Asian countries with the similar climatic conditions. The hot and humid and tropical climate of Thailand, throughout the year, is favorable for the mite, allowing it to thrive.

The worsening air pollution in Bangkok and adjacent areas over the years may have impacts on allergens, along with changes in atmospheric variables, such as CO_2_ concentration, temperature, rainfall, humidity, and wind speed and direction (18, 19). A previous study in Thailand reported that pollen is present in the air throughout the year, and grass and weed pollen, e.g., Bermuda grass (*Cynodon dactylon*), para grass (*Panicum purpurascens*), sedge (*Carex species*), and careless weed (*Amaranthus hybridus*), is more commonly found in Bangkok than tree pollen (6). These findings might explain the upwards trend in mite sensitization and persistence in the sensitization pattern for grasses (Bermuda grass, sedge, and para grass) over the last two decades, despite the fact that Bangkok is an urban center and has relatively less greenery.

Lombardi et al. (20) reported that AR patients frequently self-managed with over-the-counter medications, with a physician-based diagnosis made in only 60% of patients. The next-generation ARIA care pathway stresses self-care and self-medication with certain over-the-counter drugs, enabling the physician to step-up or step-down the treatment based on the VAS score (21). Nasal obstruction (74.6%), runny nose (63.7%), and nasal itchiness (61.5%) were the three primary symptoms present in our AR patients. Based on our findings, the majority of these symptoms are present in patients with moderate-to-severe AR, irrespective of whether it is intermittent or persistent. These findings suggest that symptom severity matters more than the symptom longevity for moderate-to-severe disease and reiterates the importance of self-care and the self-medicate care pathway.

Supporting the concept of “one airway-one disease”, 26% of AR patients in our study had cough, which re-emphasizes the fact that 40% of AR patients will also have concurrent bronchial asthma (22). In addition, 13.9% of moderate-to-severe persistent AR patients had cough, while only 6% of moderate-to-severe intermittent AR patients had cough.

Among the total number of patients recruited in our study (*n* = 366), 64.5% were women, while only 35.5% were men. However, no significant gender discrepancies were observed in terms of mean age, mean duration of symptoms, and mean age of onset of AR. The female predominance might be attributed to the better health awareness among women, as suggested in a previous study (4).

Visual analog scale is a simple and quantitative method that can be used for the quantitative evaluation of the severity of AR and for monitoring the efficacy of therapeutic interventions (23). The next-generation ARIA guideline uses an algorithm based on the VAS scores for the selection of pharmacotherapy for AR patients and to determine whether the treatment should be stepped up or stepped down depending on the status of disease control (24). In fact, the phase 3 ARIA initiative is based on Mobile Airways Sentinel Network, which aimed to initiate digitally enabled, integrated, person-centered care for rhinitis and asthma multi-morbidity using real-world evidence (25). The real-life-integrated care pathway has been adapted into the German healthcare system. An algorithm suitable for the German healthcare system using the VAS was devised and digitalised to step-up or step-down AR treatment (21). In Thailand, no previous study has reported the association between VAS scores and AR severity. Our study revealed a significant association between VAS scores and AR severity, which means that VAS can be used as a guide in the treatment and follow-up of our AR patients. The findings from our study are an important step toward the implementation of the real-world-integrated care pathway in the Thai population for the management of AR. However, in the present study, we did not assess the changes in VAS scores after initiation of treatment. Hence, further research is needed to support our findings.

A previous study in Thailand using the Thai version of the SF-36 questionnaire reported that AR patients had significantly impaired QoL scores compared with healthy individuals in all aspects, except the social functioning dimension. The same study also reported that the Rcq-36 questionnaire showed a higher correlation with the symptom scores compared with the SF-36 questionnaire, additionally including information on sleep and productivity (6). In our study, the analysis of both SF-36 and Rcq-36 questionnaires to assess the QoL showed the impact on all domains of health. The comparison of each domain of health with various categories of AR revealed that the QoL score became poorer as the severity of AR increased, which was significant (*p* > 0.05). Bodily pain, general health, and vitality were the most affected domains in the SF-36 questionnaire. Meanwhile, rhinitis symptoms, sleep, and emotions were the most affected domains in the analysis of the disease-specific questionnaire (Rcq-36). The findings from our study reaffirm that AR continues to be significantly affecting the QoL of the Thai population. Adapting and advocating the next-generation ARIA guideline with the use of real-world evidence and an integrated care pathway in the Thai population may fill the gap and adequately address the needs of AR patients.

Ciprandi and Cirillo (2) reported that the severity of symptoms was higher in poly-sensitized patients than in mono-sensitized patients. A previous study conducted in the Italian population suggested that mono-sensitization and poly-sensitization constitute two different phenotypes of AR. A similar study conducted in Malaysia demonstrated that sensitization to two or more aeroallergens was significantly associated with moderate-to-severe persistent AR (26). Previous studies from Thailand reported that all of the clinical parameters significantly affected the QoL of patients (9, 22), but did not mention the effect of sensitization status. A recent study reported that sensitization status did not show a significant association with QoL (4), but this study did not mention the effect of the number of sensitization on disease severity or QoL. In our study, 83.1% of the AR patients exhibited poly-sensitization, while only 16.9% exhibited mono-sensitization. The chi-square test between ARIA classification of AR and number of sensitizations showed no significant association (*p* = 0.49). There may be several factors that can have an effect on the clinical severity of AR due to the number of sensitizing aeroallergens. This should be addressed in future research. Similarly, our study also revealed that the type of aeroallergen and number of sensitizations had no association with VAS score and TNSS.

Our study has some limitations. It was retrospective in nature and only used a 2-year dataset. Our findings would have been better supported if we had assessed the significance of the changes in VAS scores and QoL after the initiation of treatment. In addition, due to the prevailing coronavirus disease 2019 pandemic, only a few SPTs were performed from March to May 2020.

## Conclusion

Mite and sedge remain the most common sensitizing indoor and outdoor aeroallergens in adult Thai patients with AR. The VAS scores are significantly associated with AR severity. VAS can be used as a quick and reliable tool in adult Thai patients with AR to monitor and step-up or step-down treatment. AR has a significant impact on the QoL of adult Thai patients, and the severity of AR is not associated with the number and type of aeroallergen.

## Data Availability Statement

The original contributions presented in the study are included in the article/supplementary material, further inquiries can be directed to the corresponding authors.

## Ethics Statement

The studies involving human participants were reviewed and approved by Siriraj Institutional Review Board. Certificate of Approval No. Si 296/2019. Written informed consent for participation was not required for this study in accordance with the national legislation and the institutional requirements.

## Author Contributions

PK: data collection, methodology, formal analysis, project administration, designed and wrote the first draft of the manuscript, and writing—review and editing. BP: data curation, statistical analysis, visualization, and writing—review and editing. KT: data collection and curation. PT: conceptualization, methodology, project administration, supervision, and editing. All authors contributed to the article and approved the submitted version.

## Conflict of Interest

The authors declare that the research was conducted in the absence of any commercial or financial relationships that could be construed as a potential conflict of interest.

## Publisher's Note

All claims expressed in this article are solely those of the authors and do not necessarily represent those of their affiliated organizations, or those of the publisher, the editors and the reviewers. Any product that may be evaluated in this article, or claim that may be made by its manufacturer, is not guaranteed or endorsed by the publisher.
